# Supplementary material for: Synthetic DNA Delivery of an Engineered Arginase Enzyme Can Modulate Specific Immunity In Vivo
Source: Mol Ther Methods Clin Dev. 2020 Jun 1;18:652–63. doi: 10.1016/j.omtm.2020.05.025 (PMC7406982; doi:10.1016/j.omtm.2020.05.025)
Supplement: Document S1. Figure S1 [file mmc1.pdf]

OMTM, Volume *18*

Supplemental Information

Synthetic DNA Delivery of an Engineered Arginase Enzyme Can Modulate Specific Immunity *In Vivo*

Makan Khoshnejad, Alfredo Perales-Puchalt, Yaya Dia, Peng Xiao, Ami Patel, Ziyang Xu, Xizhou Zhu, Kun Yun, Ishana Baboo, Rehman Qureshi, Laurent Humeau, Kar Muthumani, and David B. Weiner

Supplementary Figures:

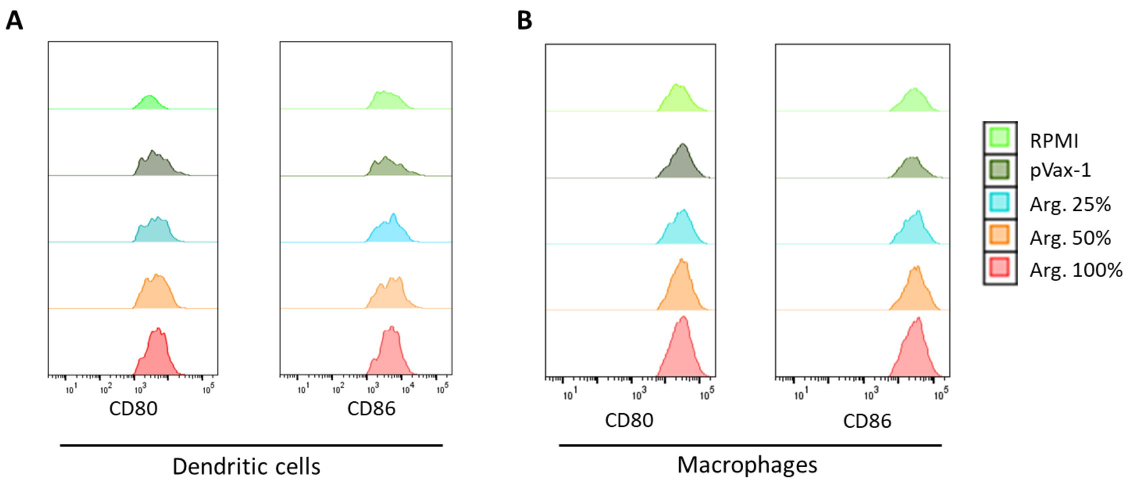

Supplementary Figure 1. Flow cytometry analysis of costimulatory molecules on bone-marrow derived dendritic cells and macrophages. Overlay histograms of co-stimulatory molecule expression in bone-marrow derived (A) dendritic cells and (B) macrophages.
