# Supplementary material for: Synthetic DNA Delivery of an Engineered Arginase Enzyme Can Modulate Specific Immunity In Vivo
Source: Mol Ther Methods Clin Dev. 2020 Jun 1;18:652–63. doi: 10.1016/j.omtm.2020.05.025 (PMC7406982; doi:10.1016/j.omtm.2020.05.025)
Supplement: Document S2. Article plus Supplemental Information [file mmc2.pdf]

Molecular Therapy
Methods & Clinical Development
Original Article

Synthetic DNA Delivery of an Engineered Arginase Enzyme Can Modulate Specific Immunity *In Vivo*

Makan Khoshnejad,[1] Alfredo Perales-Puchalt,[1] Yaya Dia,[1] Peng Xiao,[1] Ami Patel,[1] Ziyang Xu,[1,2] Xizhou Zhu,[1] Kun Yun,[1] Ishana Baboo,[1] Rehman Qureshi,[3] Laurent Humeau,[4] Kar Muthumani,[1] and David B. Weiner[1]

[1]Vaccine and Immunotherapy Center, The Wistar Institute, 3601 Spruce Street, Philadelphia, PA 19104, USA; [2]Perelman School of Medicine, University of Pennsylvania, Philadelphia, PA 19104, USA; [3]Center for Systems and Computational Biology, The Wistar Institute, 3601 Spruce Street, Philadelphia, PA 19104, USA; [4]Inovio Pharmaceuticals, Inc., Plymouth Meeting, PA 19462, USA

Arginase is a complex and unique enzyme that plays diverse roles in health and disease. By metabolizing arginine, it can shape the outcome of innate and adaptive immune responses. The immunomodulatory capabilities of arginase could potentially be applied for local immunosuppression or induction of immune tolerance. With the use of an enhanced DNA delivery approach, we designed and studied a DNA-encoded secretable arginase enzyme as a tool for immune modulation and evaluated its immunomodulatory function *in vivo*. Strong immunosuppression of cluster of differentiation 4 (CD4) and CD8 T cells, as well as macrophages and dendritic cells, was observed *in vitro* in the presence of an arginase-rich supernatant. To further evaluate the efficacy of DNA-encoded arginase on *in vivo* immunosuppression against an antigen, a cancer antigen vaccine model was used in the presence or absence of DNA-encoded arginase. Significant *in vivo* immunosuppression was observed in the presence of DNA-encoded arginase. The efficacy of this DNA-encoded arginase delivery was examined in a local, imiquimod-induced, psoriasis-like, skin-inflammation model. Pretreatment of animals with the synthetic DNA-encoded arginase led to significant decreases in skin acanthosis, proinflammatory cytokines, and costimulatory molecules in extracted macrophages and dendritic cells. These results draw attention to the potential of direct *in vivo*-delivered arginase to function as an immunomodulatory agent for treatment of local inflammation or autoimmune diseases.

INTRODUCTION

The arginine metabolism has emerged as a critical regulator of innate and adaptive immune responses. Arginine metabolism plays a major role in the function of immune cells, such as T cells, macrophages, monocytes, dendritic cells, polymorphonuclear cells, and myeloid-derived suppressor cells (MDSCs).[1,2] Mammals have two isoforms of arginase enzyme: arginase I and II.[3–5] Arginase I is located in the hepatocytes, where it functions in the urea cycle by converting arginine to urea and ornithine. Arginase II is extrahepatic with primary expression in kidney and prostate, where it regulates intracellular arginine/ornithine levels. There are two enzymes that compete for the common substrate arginine: arginase and nitric oxide synthetase (NOS). Dysregulated activity of these two enzymes can be detrimental to human health. Excessive arginase activity has been observed in hypertension, diabetes, Alzheimer's disease, stroke, and other diseases.[6–8] However, there are many beneficial effects of arginase. Arginase I expression has been associated with tissue regeneration. Ornithine, the product of arginine metabolism, is a precursor of polyamines and proline, which promote tissue regeneration by increasing collagen synthesis and proliferation.[9]

Alternatively activated (M2) macrophages and tolerogenic dendritic cells have been reported to overexpress arginase, which in turn, promotes immunosuppression. Classically activated (M1) and M2 macrophages can be characterized by their metabolism of arginine. M1 macrophages express nitric oxide synthetase, whereas the M2 macrophages express arginase.[10] Pesce et al.[11] showed that arginase I-expressing macrophages suppressed T helper 2 (Th2)-dependent inflammation and fibrosis. The immunosuppression mechanism was independent of interleukin 10 (IL-10) or transforming growth factor β1 (TGF-β1) but the result of arginine depletion leading to suppression of cluster of differentiation 4^+ ($CD4^+$) T cell proliferation and cytokine production.[11] L-arginine availability is known to regulate T cell-cycle progression. L-arginine starvation impairs expression of cyclin D3 and cdk4, leading to cell-cycle arrest. Addition of exogenous arginine was able to completely recover T cell proliferation.[12]

Downregulation of L-arginine metabolism in dendritic cells leads to induction of immune tolerance to the exogenous antigens. Simioni et al.[13] showed that treatment of bone marrow-derived dendritic cells (BMDCs) with inhibitors of arginase led to increased CD80 and CD86 costimulatory molecules in dendritic cells. Adoptive transfer of

Received 13 May 2020; accepted 27 May 2020;
https://doi.org/10.1016/j.omtm.2020.05.025.

Correspondence: David B. Weiner, Vaccine and Immunotherapy Center, The Wistar Institute, 3601 Spruce Street, Philadelphia, PA 19104, USA.
E-mail: dweiner@wistar.org

 © 2020 The Authors.
This is an open access article under the CC BY license (http://creativecommons.org/licenses/by/4.0/).

BMDCs treated with arginase inhibitors led to modulation of the immune response by mimicking the function of tolerogenic dendritic cells in immunized BALB/c mice.[13] Arginase enzyme could play a therapeutic role for autoimmune diseases. Yang et al.[14] reported that the natural polyamine, spermidine, can alleviate experimental autoimmune encephalomyelitis (EAE) by inducing inhibitory macrophages. They found that spermidine upregulates expression of arginase I in macrophages, which subsequently plays a therapeutic role in EAE.[14] Arginase, through its immunomodulatory properties, could potentially play an important therapeutic role for inflammatory and autoimmune diseases.

One promising method of DNA-encoded therapeutic delivery is the adaptive *in vivo* electroporation (EP) platform. Here, an *in vivo* EP device is used to induce low-voltage electropermeabilization to efficiently deliver the plasmid DNA to the target tissue, such as skeletal muscle or skin. The protein expression has been found to last from weeks to months depending on the dose, number of administrations, and type of protein delivered. There are a number of advantages to using a DNA-encoded therapeutic expression system, such as cost effectiveness, ease of manipulation, stability, lack of anti-vector response after repeat treatments, reduced number of administrations, and no need for cold-chain distribution.[15–17] The cost effectiveness of this technology could also alleviate disparity across economic and racially disadvantaged groups who cannot afford the high cost of protein-based therapeutics.

Here, we developed a DNA-encoded secretable murine arginase and evaluated its immunomodulatory roles *in vitro* and *in vivo*. The immunosuppressive potential of DNA-encoded arginase was evaluated in human CD4 and CD8 T cells, as well as mouse BMDCs and macrophages. Marked immunosuppression was observed in T cells, dendritic cells, and macrophages. Further evaluation of the utility of DNA-encoded arginase on *in vivo* immunosuppression in mice showed significant decreases in interferon γ (IFN-γ)-secreting cells, as well as decreases in activated CD8 T cells. An imiquimod-induced, psoriasis-like, skin-inflammation model was used to evaluate the efficacy of DNA-encoded arginase in local inflammation. Significant decreases in local inflammatory cytokines and costimulatory molecules in dendritic cells and macrophages were observed. These results demonstrate the potential of synthetic DNA-encoded arginase as an immunomodulatory agent for potential treatment of local inflammation or autoimmune diseases.

RESULTS

In Vitro Expression and Functional Activity of DNA-Encoded Arginase

DNA-encoding enzyme plasmid was designed to encode secretable murine arginase enzyme. The highly efficient immunoglobulin E (IgE) leader sequence was inserted on the 5′ end of the arginase sequence. The transgene was subcloned into the corresponding restriction enzyme site in the pVax-1 mammalian expression vector (Figure 1A). The secretable murine arginase plasmid was used to metabolize L-arginine to L-ornithine and urea (Figure 1B). The production of enzymes was confirmed *in vitro* through the transient transfection of 293T cells. Western blot analysis (Figure 1C) was performed cellular supernatant from 293T cells transfected with secretable arginase plasmids. A band near the 38-kDa marker, corresponding to the monomer of the arginase enzyme (34.8 kDa subunit), was observed with the denaturing western blot under reducing conditions. The expression of arginase was also validated by immunofluorescence microscopy (Figure 1D) in 293T cells, and staining with anti-murine arginase (mArginase) antibodies. Strong arginase expression was observed at 48 h post-transfection with the arginase-transfected cells, with no expression observed in the pVax-1 control-transfected cells. The arginase enzyme activity (Figure 1E) was measured using the QuantiChrom Arginase Assay on supernatant samples of transfected 293T cells. Significantly higher enzyme activity was observed at 24 h, 48 h, and 72 h post-transfection in the arginase-transfected cells when compared to pVax-1-transfected cells, with a high of around 135.7 U/L at 72 h.

Immunosuppressive Capability of DNA-Encoded Arginase Enzyme on T Cells, Macrophages, and Dendritic Cells *In Vitro*

In vitro T cell suppression by arginase was evaluated on human T cells by staining with CellTrace carboxyfluorescein diacetate succinimidyl ester (CSFE) staining solution, followed by incubation with the human CD3/CD28 T cell activator and IL-2 for 96 h. Flow cytometry was performed analyzing the proliferation rate of CSFE-labeled CD8 and CD4 T cells (Figures 2A and 2B). A different percentage of arginase-rich supernatant from 293T-transfected cells was used, including T cells in RPMI media as the control. Drastic suppression of both CD4 and CD8 T cell proliferation was observed with increasing the arginase concentration. The proliferation indexes (Figures 2C and 2D) for both CD4 and CD8 T cells were the lowest with 100% arginase-rich supernatants, reaching near-complete suppression of proliferation. The enzyme activity of the 100% arginase-rich supernatant was confirmed using an arginase enzyme activity assay, reaching around 96.3 U/L.

To further investigate effects of DNA-encoded arginase on the functional activity of immune cells, we generated BMDCs (Figures 2F–2H) and macrophages (Figures 2I–2K) and incubated them with supernatants from various percentages of arginase-transfected 293T cells. 96 h post-incubation, the cells were evaluated by flow cytometry and the electrochemiluminescence Meso Scale Discovery (MSD) proinflammatory detection multiplex assay. The flow cytometry data gated for dendritic cells, as well as the macrophages, demonstrated a dose-dependent decrease in the costimulatory molecules CD80 and CD86. The greatest decrease in costimulatory molecules was observed with the 100% arginase-enriched supernatant. The cytokines in the supernatants from the BMDCs and macrophages were analyzed using MSD electrochemiluminescence, with IL-6 and tumor necrosis factor α (TNF-α) showing significant dose-dependent decreases.

Molecular Pathways Underlying Changes in Dendritic Cells Following Treatment with DNA-Encoded Arginase

To study the changes in the molecular pathway of dendritic cells after incubation with arginase, normal human dendritic cells (Lonza) were

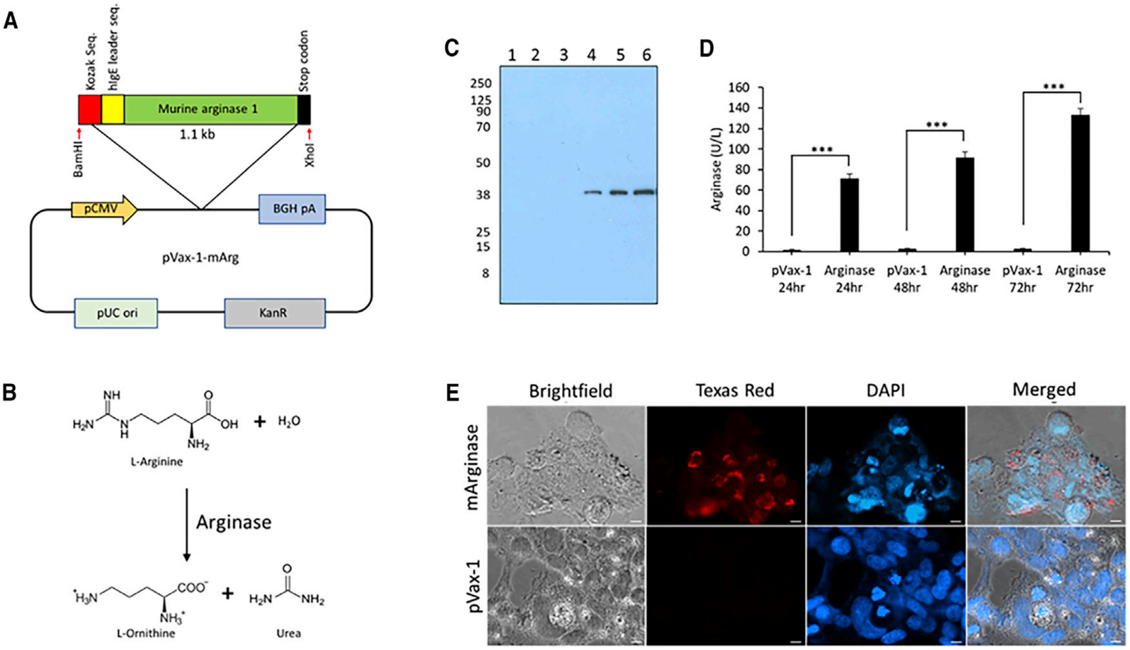

Figure 1. *In Vitro* Characterization of DNA-Encoded Murine Arginase

(A) Illustration of a secretable murine arginase (Arg.) I transgene with an IgE leader sequence incorporated in the pVax-1 mammalian vector. (B) Schematic of the Arg. metabolism by Arg. enzyme. (C) Denaturing western blot of supernatants from Arg.- or pVax-1-transfected cells. Lanes: 1, pVax-1 supernatant (sup.) 24 h; 2, pVax-1 sup. 48 h; 3, pVax-1 sup. 72 h; 4, Arg. sup. 24 h; 5, Arg. sup. 48 h; and 6, Arg. sup. 72 h. (D) Arg. enzyme activity assay performed on the supernatant from Arg.-transfected 293T cells at different time points. (E) Immunofluorescence staining of 293T cells transfected with murine Arg. plasmids. Cells were fixed 48 h after transfection. Cells were stained with anti-mouse Arg. primary antibody, anti-rabbit IgG-FITC, and DAPI nuclear stain. pVax-1-transfected cells were used as a negative control. Data are expressed as ±SEM (n = 3). Statistical differences were measured using one-way ANOVA test (*p < 0.05, **p < 0.01, ***p < 0.001; n.s., not significant).

cultured *in vitro* in the presence of IL-4 and granulocyte macrophage colony-stimulating factor (GM-CSF) for 4 days, followed by incubation for 48 h with or without the arginase-rich supernatant from 293T-transfected cells. Cells were harvested and analyzed for their mRNA expression profile using Nanostring nCounter Human Immunology Panel (Figure 3). Hierarchical clustering (Figure 3A) showed significant changes in mRNA profiles of dendritic cells between the pVax-1 control supernatant and arginase-rich supernatant. All of the arginase-rich supernatant-treated dendritic cell replicates clustered together showing similar changes in gene expression, as compared to the pVax-1 control-treated cells that clustered apart. As shown in the volcano plots (Figure 3B) comparing dendritic cells treated with the arginase-rich supernatant versus pVax-1 control supernatant, the genes that were significantly upregulated in the glutamine synthetase-treated groups were FN1, CLEC5A, TNFSF13B, CD14, CCL24, CCR1, CAMP, IFITM1, IFIT2, and FCGR2A/C. Fibronectin 1 (FN1) is involved in cell migration, adhesion, and growth. In a paper by Nikolic et al.[18], studying the differential transcriptome between tolerogenic versus inflammatory dendritic cells, FN1 was among one of the genes found to be higher in expression in tolerogenic dendritic cells. Other genes, such as CLEC5A, are a C-type lectin expressed by myeloid lineage cells. CAMP and CD14 genes have also been found to be upregulated in vitamin D3 or dexamethasone-tolerized dendritic cells, respectively. Most other genes were involved in chemotaxis or inflammatory responses. Genes that were significantly downregulated in the arginase-treated dendritic cells include ABCB1, AIRE, and C1S. ABCB1 is a transporter protein involved in dendritic cell migration. AIRE overexpression in dendritic cells has been reported to be involved in peripheral tolerance; however, a reduction was observed here. Overall, no activation of key tolerogenic pathways was detected in dendritic cells following treatment with the arginase-rich supernatant. It is possible that other pathways could be involved in suppressing the dendritic cells' immune response observed.

Expression Kinetics and Sustainability of DNA-Encoded Arginase Enzyme *In Vivo*

Intramuscular expression of DNA-encoded secreted arginase was evaluated in 6- to 8-week-old female C57BL/6 mice. Enzyme expression was confirmed by immunohistochemistry in the mouse muscle tissue of the treated mice. As shown in Figure 4A, immunostaining with anti-mouse arginase antibody-horseradish peroxidase (HRP) indicated positive browning in the mouse tibialis anterior muscle section of the DNA-encoded arginase-treated group, as compared to no browning in the control pVax-1 group. Duration of arginase enzyme in the serum of DNA-electroporated versus intravenously administered enzyme was analyzed. Intravenously administered recombinant murine arginase enzyme (1 mg/kg dose) cleared from circulation very

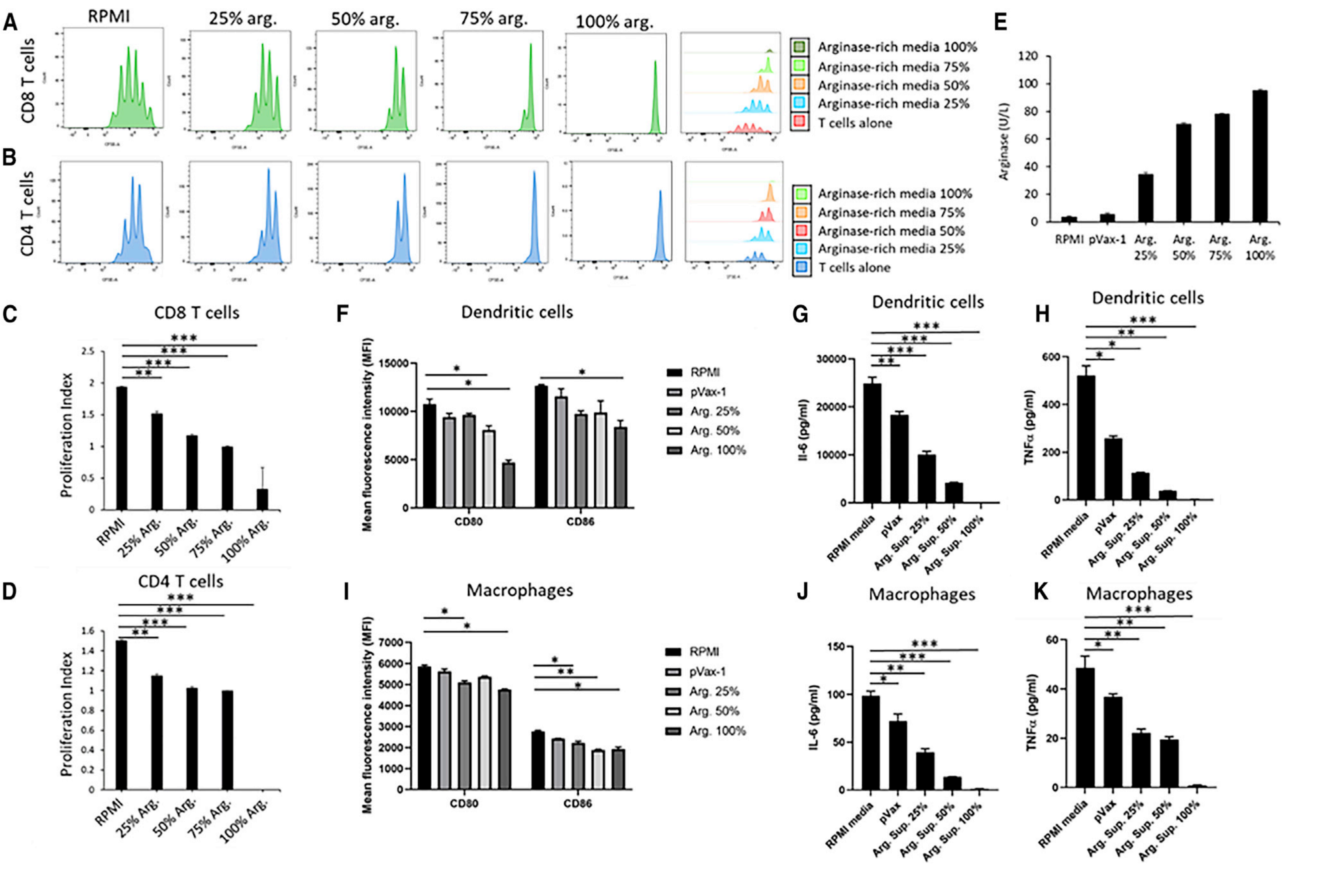

Figure 2. Evaluation of DNA-Encoded Arg. on Immunosuppression *In Vitro*

(A and B) *In vitro* T cell suppression by Arg. was evaluated on human T cells by staining with CellTrace CSFE staining solution, followed by incubation with human CD3/CD28 T cell activator and IL-2 for 96 h in the presence or absence of the Arg.-rich supernatant. Flow cytometry was performed analyzing the proliferation rate of CSFE-labeled CD8 (A) and CD4 (B) T cells. (C and D) Representative proliferation indexes for human (C) CD8 and (D) CD4 T cells treated with different percentages of Arg.-rich supernatants. (E) Arg. enzyme activity in RPMI media and supernatant samples from pVax-1- or Arg.-transfected 293T cells. A different percentage of Arg.-enriched supernatant represents the percent mixture of the Arg.-enriched supernatant with RPMI media. (F–H) Evaluation of (F) costimulatory molecules and (G and H) proinflammatory cytokines in bone marrow-derived dendritic cells (BMDCs) treated with Arg. BMDCs were generated by culturing wild-type mouse bone marrow for 8 days with 20 ng/mL GM-CSF, followed by reculturing in 24-well plates for 48 h in the presence or absence of Arg.-enriched supernatants. BMDC gated for CD11c and MHCII and costimulatory molecules CD80 and CD86. MSD electrochemiluminescence analysis of (G) IL-6 and (H) TNF-α levels was performed on supernatants, after 48 h incubation with Arg.-enriched supernatants. (I–K) Evaluation of (I) costimulatory molecules and (J and K) proinflammatory cytokines in macrophages treated with Arg. Macrophages were generated by culturing wild-type mouse bone marrow for 8 days with 20 ng/mL M-CSF, followed by reculturing in 24-well plates for 48 h in the presence or absence of Arg.-enriched supernatants. Macrophages were gated for F4/80 and CD11b and costimulatory molecules CD80 and CD86. MSD electrochemiluminescence analysis of (J) IL-6 and (K) TNF-α levels in supernatants, after 48h incubation with Arg.-enriched supernatants. Data are expressed as ±SEM (n = 3). Statistical differences were measured using one-way ANOVA tests (*p < 0.05, **p < 0.01, ***p < 0.001; n.s., not significant).

rapidly after 5 min following administration (Figure 4B). In contrast, the duration of the enzyme in the plasma of the DNA-electroporated group was significantly longer lasting. 200 μg of secretable arginase plasmid DNA was injected intramuscularly into the tibialis anterior muscle of 6- to 8-week-old female C57BL/6J mice, followed by intramuscular EP (i.m.-EP). Arginase enzyme activity assay was performed on mouse serum samples taken periodically for 42 days. Upon single intramuscular administration (Figure 4C) of the arginase plasmid, the greatest enzyme activity in the serum was observed on day 7, with gradual decrease afterward. Sequential administration (Figure 4D) of the plasmids on days 0, 7, and 14, enabled longer duration of the enzymes up to day 42. This demonstrates the sustainability of arginase expression over long periods using DNA EP. Sequential administration led to further enhancement in durability of arginase expression.

Induction of *In Vivo* Immunosuppression against TC1 Antigen Using DNA-Encoded Arginase

In order to evaluate the potential of the DNA-encoded arginase at *in vivo* immunosuppression against an antigen, we used a TC1 (lung cancer antigen) cancer vaccine model (Figure 5). The mice were primed by EP of 20 μg TC1 plasmid with/without different doses

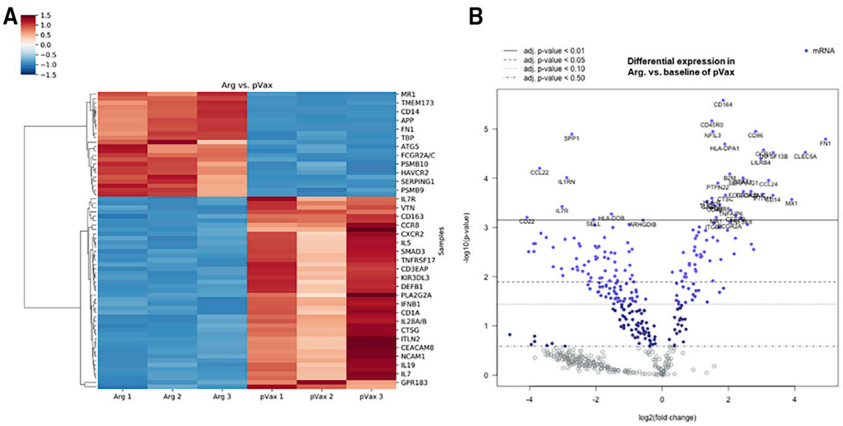

Figure 3. Changes in mRNA Expression of Human Dendritic Cells Treated with Arg.-Rich Supernatant

Normal human dendritic cells were cultured *in vitro* in the presence of IL-4 and GM-CSF for 4 days, followed by incubation for 48 h in the presence or absence of Arg.-rich supernatant. Nanostring gene-expression analysis was performed using profile using Nanostring nCounter Human Immunology Panel. (A) Hierarchical clustering of differentially expressed genes illustrating clustering of Arg.-enriched or pVax-1 control supernatant-treated human dendritic cells. (B) Volcano plots illustrating differentially expressed genes comparing pVax-1 control versus Arg.-enriched supernatant-treated human dendritic cells.

of arginase plasmid (25 μg or 50 μg) on day 0, followed with two additional boosts on days 14 and 28, and harvested on day 35. Enzyme-linked immune absorbent spot (ELISpot) analysis (Figure 5A) showed immunosuppression against the TC1 antigen, with both 25 μg and 50 μg arginase plasmid doses. The *in vivo* immunosuppression was confirmed by flow cytometry (Figures 5B–5D) with significant decreases in IFN-γ, IL-2, and TNF-α CD8 T cells. The *in vivo* immunosuppression model was repeated with even lower arginase doses (25 μg, 5 μg, 0.5 μg) to see if different doses would yield different levels of immunosuppression. ELISpot analysis (Figure 5E) showed that the 20-μg TC1 plasmid in combination with the 25-μg arginase plasmid dose still demonstrated the highest immunosuppression. No improvements in immunosuppression were achieved with doses lower than 25 μg arginase. Flow cytometry analysis (Figures 5F–5H) showed similar results, with the arginase plasmid showing reductions in IFN-γ, IL-2, and TNF-α CD8 T cells. Such an immunosuppression approach could potentially be very valuable in the development of tolerogenic vaccines for reducing immune response to the specific autoantigens for treatment of autoimmune diseases.

Skin Changes and Immunosuppressive Effects of DNA-Encoded Arginase in an Imiquimod-Induced, Psoriasis-like, Skin-Inflammation Model

In order to evaluate the efficacy of DNA-encoded arginase in a local inflammation model, an imiquimod-induced, psoriasis-like, skin-inflammation model was used. C57BL/6 mice back skin was treated with 5% imiquimod cream daily for 6 days, followed by harvest and analysis on day 7. DNA-encoded arginase was administered at different doses (25 μg or 50 μg) at day −3, using intradermal EP (ID-EP). Macroscopic views of the treated and untreated mouse skin are shown in Figure 6A. Skin scaling was observed in all imiquimod-treated mice skin samples, with or without DNA-encoded arginase. Hematoxylin and eosin (H&E) staining was performed on harvested skin samples to determine microscopic changes to the skin. Histological analysis (Figures 6B and 6C) showed significantly decreasing acanthosis with the 25-μg and 50-μg arginase plasmid doses. There was no epidermal thickening observed with the control cream or EP alone. ID-EP of the arginase enzyme at two different plasmid doses (25 μg and 50 μg dose) was performed to further investigate if arginase enzyme alone, without any imiquimod cream application, had any role on epidermal proliferation. Histological analysis showed no acanthosis on day 7 at all three plasmid doses.

Single-cell isolation was performed on harvested skin samples to analyze the local dendritic cells and macrophages activation, as well as the proinflammatory microenvironment in the treated and untreated groups. Flow cytometric analysis on dendritic cells (gated for major histocompatibility complex II^+ [$MHCII^+$] and $CD11c^+$) showed reduced costimulatory molecules CD80 and CD86 with 25 μg and 50 μg arginase plasmid doses (Figures 7A–7D). Macrophages (gated for $F4/80^+$ and $CD11b^+$) also showed a reduction in CD86 and CD80 costimulatory molecules, primarily with the 50-μg arginase plasmid dose (Figures 7E–7H). Both local macrophages and dendritic cells in the treated groups have significantly reduced costimulatory molecules. MSD electrochemiluminescence immunoassay was performed on skin tissue lysate samples (Figure 8) analyzing cytokines panel (TNF-α, IL-1β, IL-6, IL-10, IL-17A, IL-22, IL-23, and IFN-γ). Compared to the imiquimod cream alone, there was significant decreases in TNF-α, IL-6, and IL-17A. 25 μg and 50 μg arginase plasmid doses showed decreases in proinflammatory cytokine levels. These results show significant immunosuppressive properties of DNA-encoded arginase in a local, skin-inflammation model.

DISCUSSION

Arginase is a complex enzyme that plays a major role in health and disease. Metabolism of arginine by arginase can drastically alter the function of many immune cells, shaping the innate and adaptive immune responses. Arginine metabolism can modulate the function of macrophages, dendritic cells, MDSCs, polymorphonuclear cells, and T cells.[1,2] M2 macrophages have been found to express arginase I, which in turn, plays a role in dampening T cell effector responses.[10] T cells undergo cell-cycle arrest with arginine starvation.[12] Anti-tumor responses can be inhibited by arginase-expressing immune cells in the tumor microenvironment. Arginase I expression by tumor-infiltrating regulatory dendritic cells has been reported to be one of the key pathways in suppressing the $CD8^+$ T cell anti-tumor

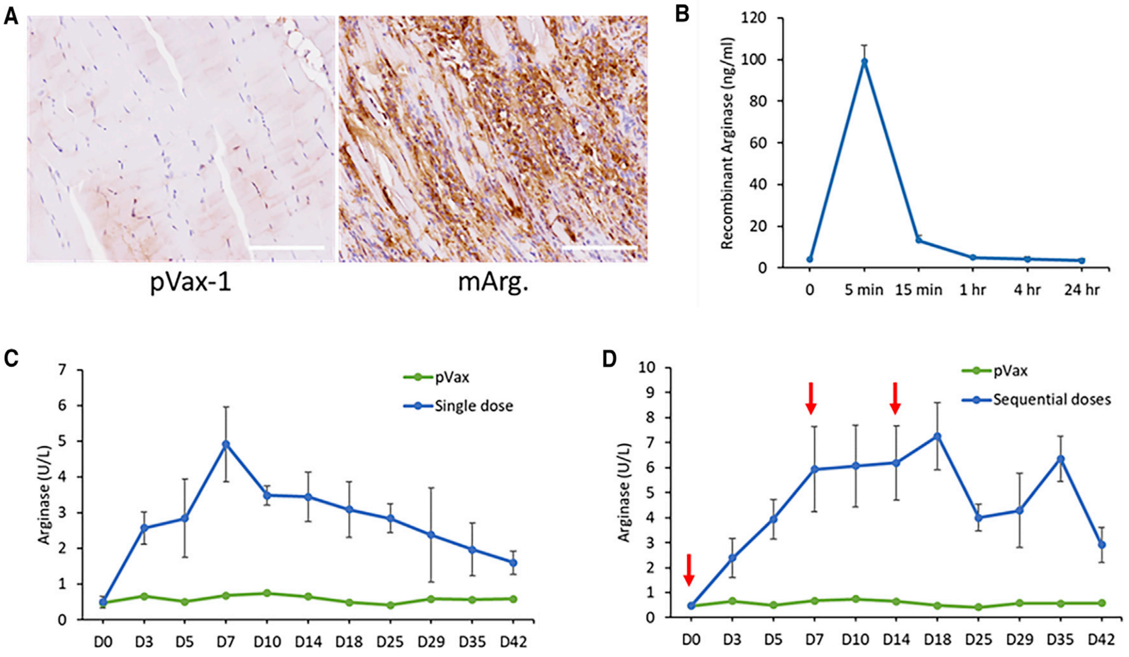

Figure 4. *In Vivo* Expression Analysis of DNA-Encoded Arg.

(A) Immunohistochemistry of Arg. using anti-mouse Arg. antibody for mouse muscle sections at Day 3. C57BL/6 mice were administered 150 μg of Arg. plasmid in the tibialis anterior muscle. Scale bars, 100 μm. (B) Quantitative ELISA analysis of intravenous administration (1 mg/kg) of recombinant murine Arg. protein in C57BL/6 mice. (C and D) Quantitative ELISA analysis of DNA-encoded Arg. following a (C) single or (D) sequential intramuscular injection of plasmids at days 0, 7, and 14 in C57BL/6 mice. Values represent mean expression in each group (n = 5) ± SEM.

response.[19,20] Some have been using arginine starvation as a strategy to directly kill tumor cells. Polyethylene glycol (PEG)ylated arginine metabolizing enzymes and arginase I have been used as anti-cancer treatment approaches for leukemia, melanoma, and hepatocellular carcinoma. Arginase I has been found to induce cell-cycle arrest, apoptosis, and autophagy in tumor cells.[21–25]

Arginine metabolism by the arginase enzyme can have major therapeutic implications in inflammatory and autoimmune diseases. Today's pharmacotherapeutics for autoimmune disease have mainly consisted of systemic immunosuppressives, which have brought about unfortunate, adverse side effects and long-term risks to the patients and are not capable of achieving long-lasting disease remission. Autoimmune diseases are generally caused by a break in tolerance of self-antigens of a range of factors, such as environmental, infections agents, or dietary factors. It is essential to re-establish tolerance toward these autoantigens by means, such as the development of tolerogenic vaccines or approaches capable of providing antigen-specific immunosuppression. Arginase could potentially be used as a therapeutic approach for local inflammation or as an immunomodulatory approach to re-establish tolerance toward the autoantigen for the treatment of autoimmune disease.

Here, we developed a DNA-encoded, EP-enhanced, immunomodulatory-secretable arginase enzyme for potential use in treatment of local inflammation or autoimmune disease. DNA-encoded secretable arginase was evaluated for efficacy at immunosuppression. An *in vitro* T cell proliferation study showed marked suppression of proliferation in both CD4 and CD8 T cells, with increasing percentages of arginase-rich supernatants. This corroborates with the studies on arginine starvation and arginase produced from myeloid cells leading to T cell suppression.

Further evaluation of DNA-encoded arginase in BMDCs and macrophages demonstrated decreases in CD80 and CD86 costimulatory molecules, as well proinflammatory cytokines TNF-α and IL-6. A TC1 cancer neoantigen vaccine model was used to determine if DNA-encoded arginase was capable of immunosuppression against an antigen. IFN-γ ELISpot analysis showed dose-dependent decreases in IFN-γ-secreting cells, as well as decreases in IL-2-, IFN-γ-, and TNF-α-positive CD8 T cells with flow cytometric analysis. Such an immunosuppression approach could potentially have implications in the treatment of autoimmune disease.

In order to evaluate the effect of DNA-encoded arginase in a local inflammation model, an imiquimod-induced, psoriasis-like, skin-inflammation model was used. There are conflicting reports on the efficacy of arginase for treatment of psoriasis; some reported detrimental effects by reduction in nitric oxide synthetase activity leading to reduced nitric oxide, which has been reported to be beneficial for treatment of psoriasis. Others have emphasized the pathophysiological role of proinflammatory cytokines in keratinocyte proliferation.

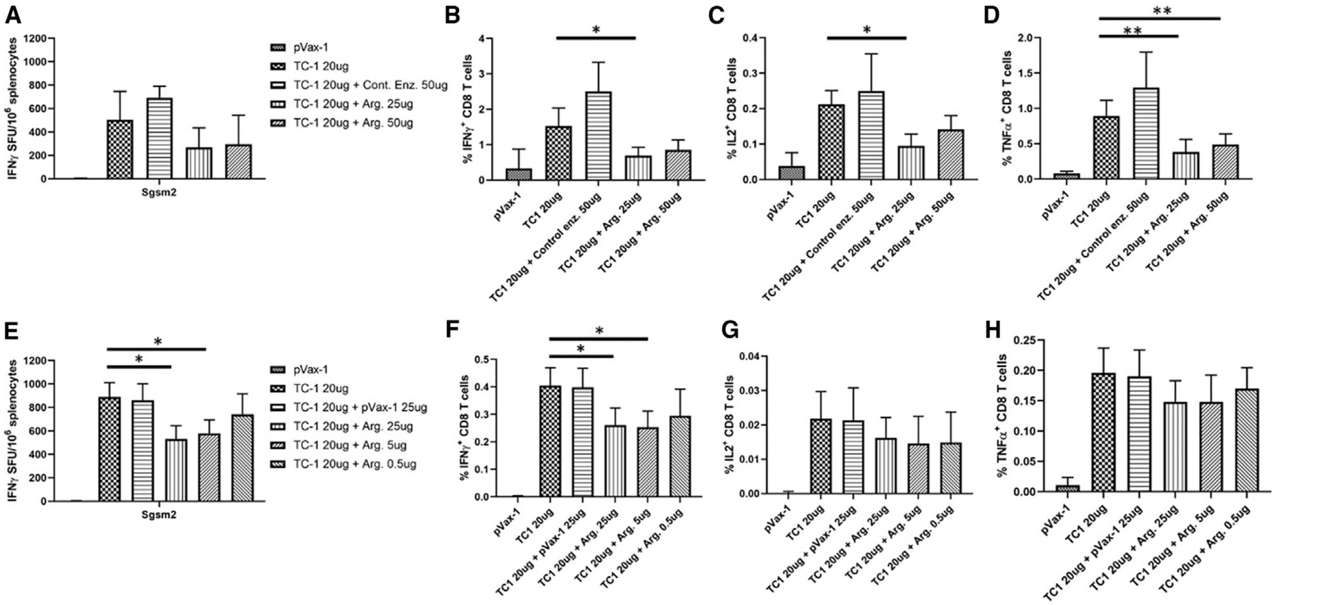

Figure 5. Induction of *In Vivo* Immunosuppression against TC1 Cancer Antigen Using DNA-Encoded Arg.
C57BL/6 mice were immunized on day 0 with 20 μg TC1 antigen or control plasmids in the presence or absence of different doses of Arg. plasmids. Two boosts were administered on days 14 and 28, with harvest on day 35. (A and E) IFN-γ ELISpot assay was performed using (A) high doses or (E) low doses of DNA-encoded Arg., measuring for IFN-γ-producing spot-forming units (SFUs) generated per 10^6 splenocytes. (B–D and F–H) Flow cytometry analysis of (B–D) high doses or (F–H) low doses of DNA-encoded Arg. to determine the frequency of $CD8^+$ T cells producing the cytokines (B and F) IFN-γ, (C and G) IL-2, and (D and H) TNF-α. Data are expressed as ±SEM (n = 5). Statistical differences were measured using one-way ANOVA test (*p < 0.05, **p < 0.01, ***p < 0.001; n.s., not significant).

Here, we evaluated arginase for resolution of local inflammation and not as a therapeutic for psoriasis. From our results, dendritic cells and macrophages isolated from the mouse skin in the imiquimod and arginase plasmid-treated groups showed significant decreases in CD80 and CD86 costimulatory molecules. The MSD electrochemiluminescence proinflammatory panel of the harvest skin lysate showed significant decreases in proinflammatory cytokines TNF-α, IL-6, and IL-17A. Histological analysis showed decreases in the epidermal skin proliferation at the 25-μg and 50-μg arginase plasmid doses. The DNA-encoded arginase pretreatment led to significant decreases in inflammation in the imiquimod-administered skin tissue.

The remarkable immunosuppressive capability of DNA-encoded arginase on dendritic cells, macrophages, and T cells, including immunosuppression against an antigen, highlights its therapeutic potential for use in local inflammation and autoimmune diseases. Through the EP-enhanced nucleic acid delivery platform, a DNA-encoded arginase combination with autoantigen could potentially be used to induce autoantigen-specific immunosuppression. This approach could potentially offer an alternative to provide long-lasting, autoantigen-specific immune tolerance and avoid or reduce the dependence on systemic immunosuppressives.

MATERIALS AND METHODS

Cell Culture

Human embryonic kidney (HEK) 293T cells were cultured in Dulbecco's modified Eagle's medium (Life Technologies, Carlsbad, CA, USA), supplemented with 10% fetal bovine serum (FBS), 100 U/mL penicillin, and 100 μg/mL streptomycin (Life Technologies, Carlsbad, CA, USA).

Arginase Plasmid Design and Construction

A secretable murine arginase construct was designed and synthesized. The murine arginase transgene bearing a highly efficient IgE leader sequence on the 5′ end to improve protein translation was synthesized and subcloned into the corresponding restriction enzyme site in the pVax-1 mammalian expression vector. Human cytomegalovirus (CMV) immediate-early promoter in the pVax-1 vector drives the expression of the arginase enzyme.

Transfection of Arginase Plasmids

HEK293T cells were transfected with arginase plasmids using Lipofectamine LTX Reagent (Thermo Fisher Scientific, Waltham, MA, USA) and following the manufacturer's protocol. Briefly, cells were seeded at a density of 5×10^5 cells/well in a 12-well plate and incubated in a 37°C incubator with 5% CO_2. Once cells reached 70% confluence, they were transfected with 1 μg arginase plasmid and incubated for a maximum of 72 h. Arginase enzyme expression and activity following transfection were analyzed by an enzyme activity assay, western blot, and immunofluorescence microscopy.

Western Blot, ELISA, and Enzyme Activity Analysis

Western blot analysis was performed on the cellular supernatant at 24 h, 48 h, and 72 h time points from 293T cells transfected with

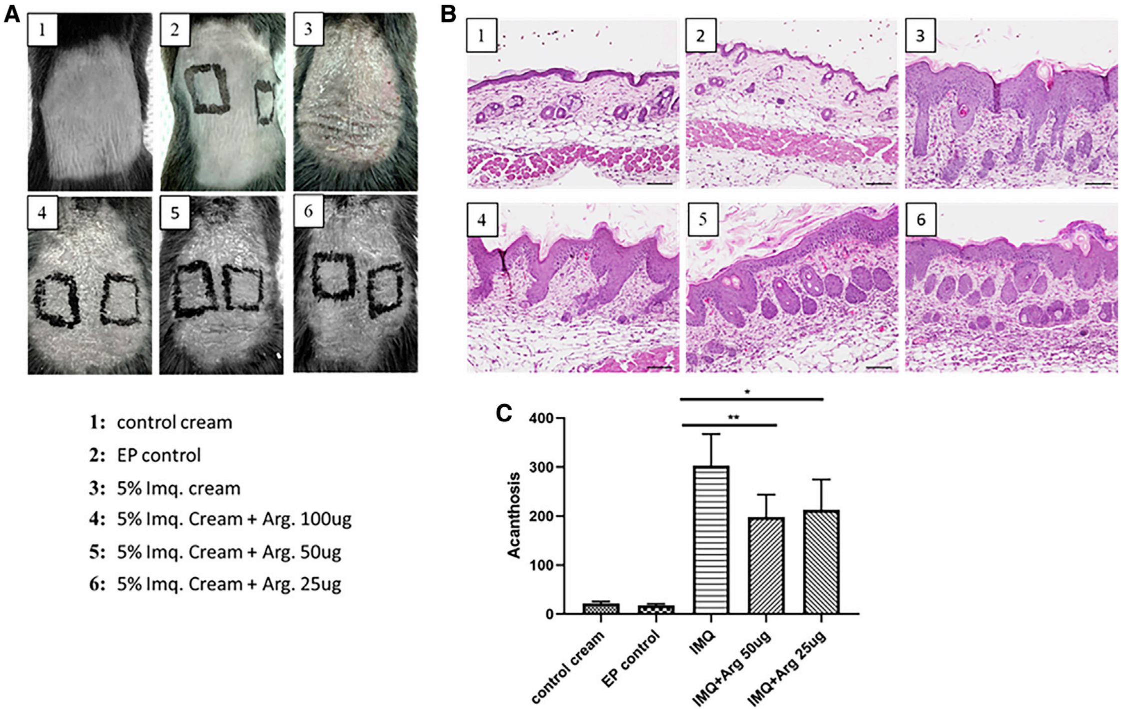

Figure 6. Changes in the Skin of Mice Following Treatment with DNA-Encoded Arg. in an Imiquimod-Induced, Psoriasis-like, Skin-Inflammation Model
C57BL/6 mice back skin was treated with 5% imiquimod cream daily for 6 days, followed by harvest and analysis on day 7. DNA-encoded Arg. was administered at different doses (25 μg or 50 μg) at day −3 using intradermal electroporation (ID-EP). (A) Macroscopic changes in the skin of the treated and untreated mice on day 7. (B and C) Hematoxylin and eosin (H&E) staining (B) and the corresponding level of acanthosis (C) of harvested skin samples at day 7. Data are expressed as ± SEM (n = 5). Statistical differences were measured using one-way ANOVA test (*p < 0.05, **p < 0.01, ***p < 0.001; n.s., not significant).

arginase plasmids. The supernatant samples were run under denaturing and reducing conditions in precast NuPAGE 10% Bis-Tris gels (Invitrogen, Carlsbad, CA, USA). The gels were transferred to the polyvinylidene fluoride (PVDF) transfer membrane (Millipore, Burlington, MA, USA) using the iBlot 2 Dry Blotting System (Life Technologies, Carlsbad, CA, USA). The membranes were blocked for 1 h in 3% nonfat dry milk in phosphate-buffered saline (PBS) with 0.1% Tween 20 (Sigma-Aldrich, St. Louis, MO, USA). The membrane was stained with an anti-mouse arginase primary antibody (GeneTex, Irvine, CA) and an anti-rabbit IgG-HRP secondary antibody (Abcam, Cambridge, UK). The recombinant arginase enzyme was quantified by ELISA, by coating the MaxiSorp plates (Thermo Fisher Scientific, Waltham, MA, USA) with samples and the recombinant arginase protein (LifeSpan BioSciences, Seattle, WA, USA) standards and incubation overnight at 4°C. After blocking the plates in 10% FBS in PBS for 1 h at room temperature (RT), the plates were washed and incubated with anti-mouse arginase primary antibody (GeneTex, Irvine, CA, USA), 1 h at RT, followed by anti-rabbit IgG-HRP secondary antibody (Abcam, Cambridge, UK) for another hour at RT. Sample detection was performed using Sigmafast o-phenylenediamine dihydrochloride (OPD; Sigma-Aldrich, St. Louis, MO, USA) substrate solution. Optical density was measured at 450 nm using a BioTek Synergy 2 Plate Reader (BioTek, Winooski, VT, USA).

Arginase enzyme activity was measured using a QuantiChrom Arginase Assay Kit (BioAssay Systems, Hayward, CA, USA), according to the manufacturer's protocol. Enzyme activity was quantified on serum, muscle tissue, or cellular supernatant of transfected cells.

Immunofluorescence Analysis

Arginase plasmid was transfected in 293T cells using Lipofectamine LTX Reagent, as described above. 2 days after transfection, cells were fixed and permeabilized with 4% paraformaldehyde in PBS and 0.5% Triton X-500. Cells were stained with anti-mouse arginase primary antibody (GeneTex, Irvine, CA) and anti-rabbit-IgG-Texas Red (Abcam, Cambridge, UK) for 1 h. Cell were washed with PBS and stained with 4′,6-diamidino-2-phenylindole (DAPI) nuclear stain (Thermo Fisher Scientific, Waltham, MA, USA). Cells were imaged on a Nikon TE2000 inverted microscope imaging system.

Immunohistochemistry of Mouse Muscle

Immunohistochemistry of the mouse muscle arginase expression was performed by The Wistar Institute Histotechnology Facility. Briefly, the arginase and pVax-1 control plasmid-treated anterior tibialis muscle sections were resected and embedded in paraffin. Antigen retrieval reagent-treated, paraffin-embedded samples were deparaffinized. Acetone was used to fix the slides, followed by washing with

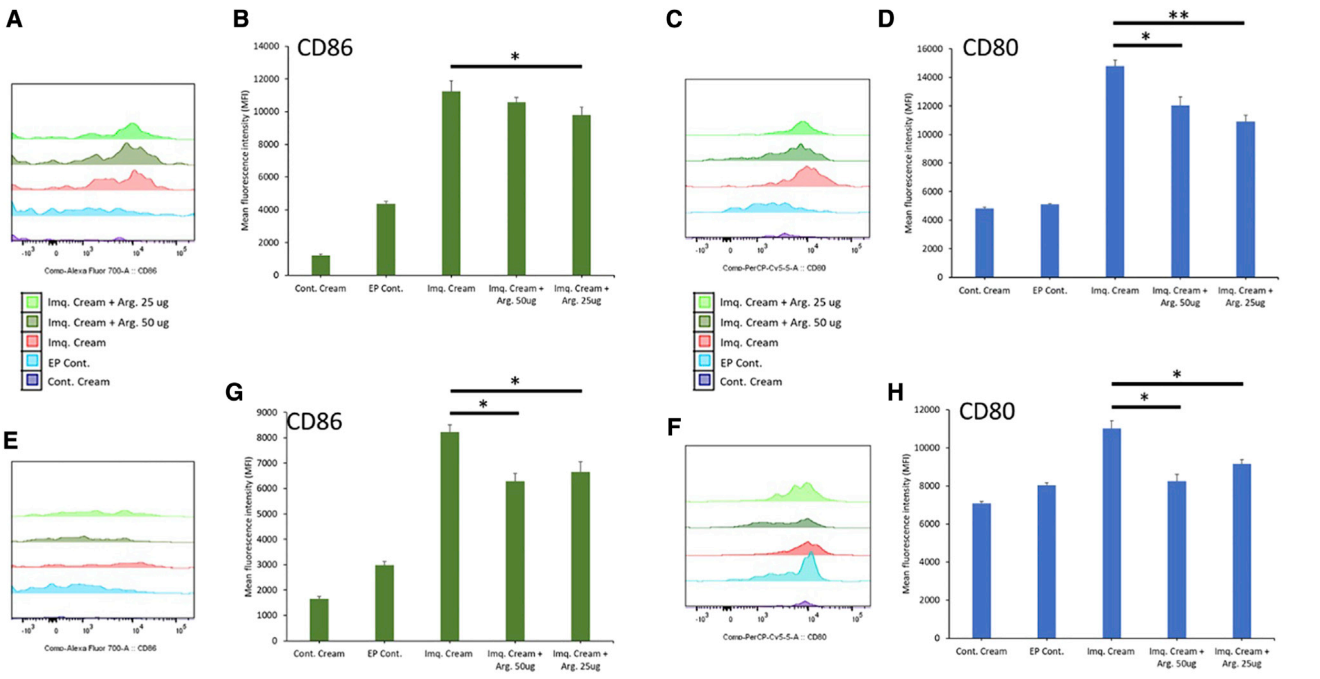

Figure 7. Flow Cytometric Analysis of the Costimulatory Molecules in Skin Dendritic Cells and Macrophages from Mice Treated with DNA-Encoded Arg. in an Imiquimod-Induced, Psoriasis-like, Skin-Inflammation Model

(A–D) Histograms (A and C) and mean fluorescence intensity (MFI) (B and D) of CD86 (A and B) and CD80(C and D) costimulatory molecules on dendritic cells gated for MHCII and CD11c. (E–H) Histograms (E and F) and MFI (G and H) of CD86 (E and G) and CD80 (F and H) costimulatory molecules on macrophages gated for F4/80 and CD11b. Data are expressed as ±SEM (n = 5). Statistical differences were measured using one-way ANOVA test (*p < 0.05, **p < 0.01, ***p < 0.001; n.s., not significant).

PBS. The slides were blocked and stained with anti-mouse arginase primary antibody and anti-rabbit IgG-HRP secondary antibody.

In Vitro T Cell-Suppression Assay

Human T cells were stained with CellTrace CSFE staining solution (Thermo Fisher Scientific, Waltham, MA, USA). Cells were then incubated with Invitrogen Dynabeads Human CD3/CD28 T Cell Activator (Thermo Fisher Scientific, Waltham, MA, USA) and IL-2 (Thermo Fisher Scientific, Waltham, MA, USA) for 96 h in the presence or absence of arginase-rich supernatant. Different percentages of supernatants from arginase-transfected 293T cells were used. Flow cytometer was used for gating for CD4 and CD8 T cells, as well as 488 nm excitation and emission for the CFSE fluorescence. Antibodies used for T cell gating included allophycocyanin (APC) anti-CD3 (BioLegend, San Diego, CA, USA), phycoerythrin (PE) CD4 (BioLegend, San Diego, CA, USA), and APC-Cy7 anti-CD8 (BioLegend, San Diego, CA, USA). The experiment was performed in triplicates.

ELISpot Assay

A mouse IFN-γ ELISpot^{PLUS} (Mabtech, Stockholm, Sweden) assay was used, according to the manufacturer's protocol. Briefly, 2×10^5 splenocytes from the TC1, with or without arginase or pVax-1 control-immunized mice, were added per well and incubated at 37°C in 5% CO_2 for 18 h. Cells were stimulated with TC1 antigen peptide pools (1 μg/mL). Media alone were used as a negative control. As the positive control, media with Cell Activation Cocktail (BioLegend, San Diego, CA, USA) containing pre-mixed phorbol 12-myristate-13-acetate (PMA) and ionomycin (positive control) were used. 5-bromo-4-chloro-3-indolyl-phosphate/nitro blue tetrazolium (BCIP/NBT) color-development substrate (R&D Systems, Minneapolis, MN, USA) was used for plate spot formation. Quantification of the spot-forming units (SFUs) was performed by an automated ELISpot Reader (CTL, Shaker Heights, OH, USA).

Flow Cytometry

For the TC1 vaccine study, 2×10^6 single-cell splenocytes per well were added to a U-bottom, 96-well plate (Thermo Fisher Scientific, Waltham, MA). Cells were washed with fluorescence-activated cell sorting (FACS) buffer containing 3% bovine serum albumin (BSA) in PBS and stained for the surface proteins using fluorochrome-conjugated antibodies, according to the manufacturer's protocol (BD Biosciences, San Jose, CA, USA). After washing cells with FACS buffer, cells were fixed and permeabilized using BD Cytofix/Cytoperm (BD Biosciences, San Jose, CA, USA). The cells were stained with intracellular cytokines using fluorochrome-conjugated antibodies (BD Biosciences, San Jose, CA, USA). Fluorochrome-containing reagents included the LIVE/DEAD Fixable Violet Dead Cell Stain Kit (Invitrogen, Carlsbad, CA, USA), CD4 (fluorescein isothiocyanate [FITC]; eBioscience, San Diego, CA, USA), CD8α (APC-Cy7; BD

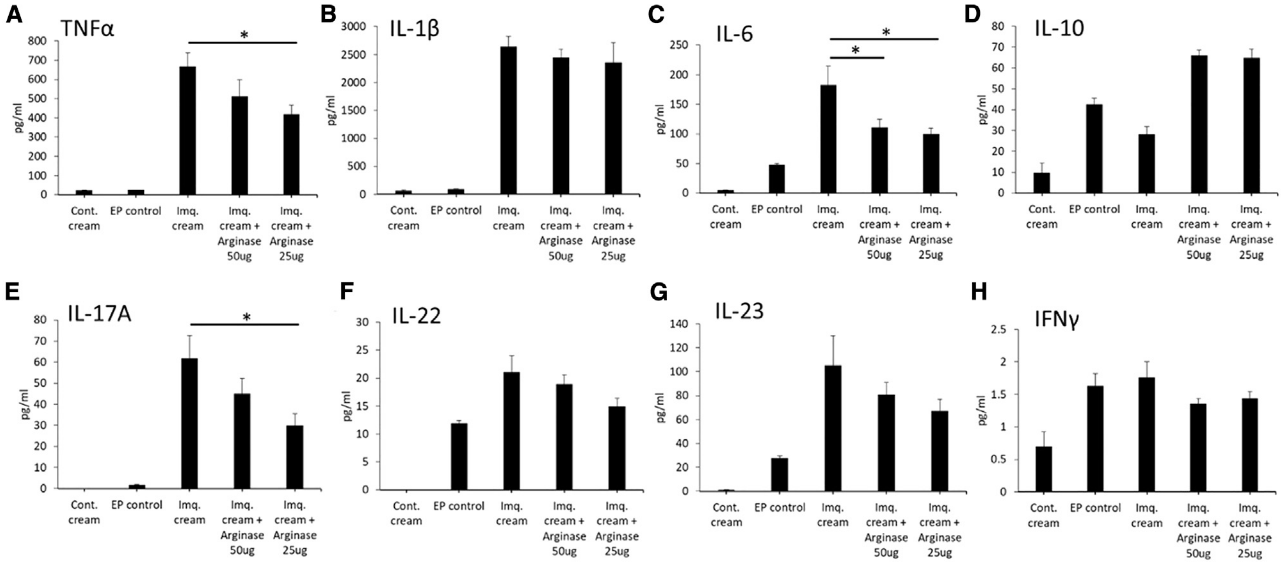

Figure 8. MSD Electrochemiluminescence Immunoassay of Skin Tissue Lysate Samples from Mice Treated with DNA-Encoded Arg. in an Imiquimod-Induced, Psoriasis-like, Skin-Inflammation Model

(A–H) Cytokine levels in skin tissue lysate samples: (A) TNF-α, (B) IL-1β, (C) IL-6, (D) IL-10, (E) IL-17A, (F) IL-22, (G) IL-23, and (H) IFN-γ. Data are expressed as ±SEM (n = 5). Statistical differences were measured using one-way ANOVA test (*p < 0.05, **p < 0.01, ***p < 0.001; n.s., not significant).

Biosciences, San Jose, CA, USA), IFN-γ (APC; BioLegend, San Diego, CA, USA), TNF-α (PE; eBioscience, San Diego, CA, USA), CD3ε (PerCP/Cy5.5; BioLegend, San Diego, CA, USA), and IL-2 (PE-Cy7; eBioscience, San Diego, CA, USA). For the evaluation of macrophage and dendritic cell expression of costimulatory molecules, mouse BMDCs were grown in 20 ng/mL mouse GM-CSF (mGM-CSF; Thermo Fisher Scientific, Waltham, MA, USA) for dendritic cell differentiation or 20 ng/mL mGM-CSF (Thermo Fisher Scientific, Waltham, MA, USA) for macrophage differentiation for 8 days, with media replenishments on days 3 and 6. On day 8, 5×10^5 cells were plated in a 24-well plate and incubated with arginase-rich supernatants for 48 h. After 48 h, dendritic cells were stained with the Zombie Yellow Fixable Viability Kit (BioLegend, San Diego, CA, USA), CD11c (FITC; BioLegend, San Diego, CA, USA), MHCII (PE; Thermo Fisher Scientific, San Diego, CA, USA), CD86 (PE/Cy7; BioLegend, San Diego, CA, USA), and CD80 (PerCP/Cy5.5; BioLegend, San Diego, CA, USA). The macrophages were stained with the Zombie Yellow Fixable Viability Kit (BioLegend, San Diego, CA, USA), CD11b (BV785; BioLegend, San Diego, CA, USA), F4/80 (BV421; BioLegend, San Diego, CA, USA), CD86 (PE/Cy7; BioLegend, San Diego, CA, USA), and CD80 (PerCP/Cy5.5; BioLegend, San Diego, CA, USA). Data were collected using a LSRII flow cytometer (BD Biosciences, San Jose, CA, USA) and analyzed using FlowJo software.

MSD Electrochemiluminescence Assay

For the MSD electrochemiluminescence studies, the mouse V-PLEX Proinflammatory Panel 1 Kit from Meso Scale Diagnostics (Rockville, MD, USA) was used, according to the manufacturer's protocol. Plates were read on a MESO SECTOR S 600 imager and analyzed using the Discovery Workbench software. All standards and samples were measured in duplicates.

NanoString nCounter mRNA Profiling

NanoString gene-expression analysis was performed using the profile using the NanoString nCounter Human Immunology Panel (NanoString Technologies, Seattle, WA, USA). Total RNA was extracted from normal human dendritic cells cultured in the presence or absence of arginase-rich supernatant from 293T-transfected cells. RNA extraction was performed by the Wistar Genomics Core. RNA samples were profiled according to standard NanoString protocols on a NanoString nCounter Analysis System operated by the Wistar Genomics Core. Data were analyzed using NanoString nSolver software.

In Vivo Immunosuppression Study

Evaluation of the DNA-encoded TC1 cancer antigen, with or without arginase, was carried out in female 6- to 8-week-old C57BL/6J mice (n = 5 mice per group). Animal experiments were carried out in accordance with the guidelines of the National Institutes of Health (NIH) and The Wistar Institute Institutional Animal Care and Use Committee (IACUC). Mice were injected with plasmid DNA in the tibialis anterior muscles. *In vivo* EP was carried out using a CELLECTRA adaptive constant current EP device (Inovio Pharmaceuticals). Triangular 3-electrode arrays, consisting of 26-gauge solid stainless-steel electrodes, were used to deliver square-wave pulses. Two constant current pulses were delivered at 0.1 A for 52 ms/pulse, separated with a 1-s delay between pulses. Mice were given a dose of

20 μg TC1 neoantigen plasmid 1 DNA[26] in the presence of arginase (0.5, 5, 25, or 50 μg) or 20 μg of control pVax-1 or control enzyme, resuspended in water. A 30-μL volume was used at each injection site. Immunizations were performed on days 0, 14, and 28, with harvest of mice on day 35. The splenocytes were analyzed by ELISpot and flow cytometry.

Psoriasis-like Inflammation Study

An imiquimod-induced, psoriasis-like inflammation model was performed in female 6- to 8-week-old C57BL/6J mice (n = 5 mice per group). Topical administration of 62.5 mg of 5% imiquimod cream (Perrigo, Dublin, Ireland) was applied daily on the shaved back of the mouse for 6 days. Arginase plasmid DNA (25 μg or 50 μg DNA in 30 μL saline solution) was administered by ID-EP at back-skin tissue at day −3. Plasmid DNA was injected into the skin using a Mantoux injection, immediately followed by surface ID-EP, using a 4 × 4 array of probes placed lightly onto the skin, followed by pulses. Two sets of 2 pulses (at 0.1 A) were delivered. Each set of 2 pulses lasted 52 ms with a 1-s pause in between each set of pulses. Mice were harvested on day 7 for analysis by the MSD electrochemiluminescence assay, H&E staining, and flow cytometry.

Statistics

Experimental data were analyzed using one-way ANOVA. Differences were deemed significant at p values < 0.05. All graphs were prepared using GraphPad Prism 6 (GraphPad Software).

SUPPLEMENTAL INFORMATION

Supplemental Information can be found online at https://doi.org/10.1016/j.omtm.2020.05.025.

AUTHOR CONTRIBUTIONS

M.K. performed the experiments, analyzed data, and wrote the manuscript. M.K., A.P., and D.B.W. were involved in study conception and design. M.K., AP.-P., Y.D., P.X., A.P., Z.X., X.Z., K.Y., I.B., R.Q., L.H., K.M., and D.B.W. were involved in experimental design and data analysis and contributed intellectually to the research. All authors contributed to manuscript editing.

CONFLICTS OF INTEREST

L.H. is an employee of Inovio Pharmaceuticals and as such, receives salary and benefits, including ownership of stock and stock options. D.B.W. discloses grant funding, industry collaborations, speaking honoraria, and fees or stock for consulting. His service includes serving on scientific review committees and advisory boards. Remuneration includes direct payments and/or stock or stock options. K.M. reports receiving grants from Inovio and receiving consulting fees from Inovio related to DNA vaccine development. The other authors declare no competing financial interests.

ACKNOWLEDGMENTS

This work was supported by funding from Inovio Pharmaceuticals to M.K. and D.B.W.

REFERENCES

1. Rodriguez, P.C., Ochoa, A.C., and Al-Khami, A.A. (2017). Arginine metabolism in myeloid cells shapes innate and adaptive immunity. Front. Immunol. *8*, 93.
2. Patel, C.H., Leone, R.D., Horton, M.R., and Powell, J.D. (2019). Targeting metabolism to regulate immune responses in autoimmunity and cancer. Nat. Rev. Drug Discov. *18*, 669–688.
3. Ash, D.E. (2004). Structure and function of arginases. J. Nutr. *134* (10, Suppl), 2760S–2764S, discussion 2765S–2767S.
4. Di Costanzo, L., Moulin, M., Haertlein, M., Meilleur, F., and Christianson, D.W. (2007). Expression, purification, assay, and crystal structure of perdeuterated human arginase I. Arch. Biochem. Biophys. *465*, 82–89.
5. Morris, S.M., Jr. (1992). Regulation of enzymes of urea and arginine synthesis. Annu. Rev. Nutr. *12*, 81–101.
6. Caldwell, R.W., Rodriguez, P.C., Toque, H.A., Narayanan, S.P., and Caldwell, R.B. (2018). Arginase: A multifaceted enzyme important in health and disease. Physiol. Rev. *98*, 641–665.
7. Yang, Z., and Ming, X.F. (2013). Arginase: the emerging therapeutic target for vascular oxidative stress and inflammation. Front. Immunol. *4*, 149.
8. Moretto, J., Girard, C., and Demougeot, C. (2019). The role of arginase in aging: A systematic review. Exp. Gerontol. *116*, 54–73.
9. Campbell, L., Saville, C.R., Murray, P.J., Cruickshank, S.M., and Hardman, M.J. (2013). Local arginase 1 activity is required for cutaneous wound healing. J. Invest. Dermatol. *133*, 2461–2470.
10. Rath, M., Müller, I., Kropf, P., Closs, E.I., and Munder, M. (2014). Metabolism via arginase or nitric oxide synthase: Two competing arginine pathways in macrophages. Front. Immunol. *5*, 532.
11. Pesce, J.T., Ramalingam, T.R., Mentink-Kane, M.M., Wilson, M.S., El Kasmi, K.C., Smith, A.M., Thompson, R.W., Cheever, A.W., Murray, P.J., and Wynn, T.A. (2009). Arginase-1-expressing macrophages suppress Th2 cytokine-driven inflammation and fibrosis. PLoS Pathog. *5*, e1000371.
12. Rodriguez, P.C., Quiceno, D.G., and Ochoa, A.C. (2007). L-arginine availability regulates T-lymphocyte cell-cycle progression. Blood *109*, 1568–1573.
13. Simioni, P.U., Fernandes, L.G.R., and Tamashiro, W.M.S.C. (2017). Downregulation of L-arginine metabolism in dendritic cells induces tolerance to exogenous antigen. Int. J. Immunopathol. Pharmacol. *30*, 44–57.
14. Yang, Q., Zheng, C., Cao, J., Cao, G., Shou, P., Lin, L., Velletri, T., Jiang, M., Chen, Q., Han, Y., et al. (2016). Spermidine alleviates experimental autoimmune encephalomyelitis through inducing inhibitory macrophages. Cell Death Differ. *23*, 1850–1861.
15. Flingai, S., Plummer, E.M., Patel, A., Shresta, S., Mendoza, J.M., Broderick, K.E., Sardesai, N.Y., Muthumani, K., and Weiner, D.B. (2015). Protection against dengue disease by synthetic nucleic acid antibody prophylaxis/immunotherapy. Sci. Rep. *5*, 12616.
16. Muthumani, K., Block, P., Flingai, S., Muruganantham, N., Chaaithanya, I.K., Tingey, C., Wise, M., Reuschel, E.L., Chung, C., Muthumani, A., et al. (2016). Rapid and Long-Term Immunity Elicited by DNA-Encoded Antibody Prophylaxis and DNA Vaccination Against Chikungunya Virus. J. Infect. Dis. *214*, 369–378.
17. Muthumani, K., Marnin, L., Kudchodkar, S.B., Perales-Puchalt, A., Choi, H., Agarwal, S., Scott, V.L., Reuschel, E.L., Zaidi, F.I., Duperret, E.K., et al. (2017). Novel prostate cancer immunotherapy with a DNA-encoded anti-prostate-specific membrane antigen monoclonal antibody. Cancer Immunol. Immunother. *66*, 1577–1588.
18. Nikolic, T., et al. (2017). Differential transcriptome of tolerogenic versus inflammatory dendritic cells points to modulated T1D genetic risk and enriched immune regulation. Genes and Immunity *3*, 176–183.
19. Norian, L.A., Rodriguez, P.C., O'Mara, L.A., Zabaleta, J., Ochoa, A.C., Cella, M., and Allen, P.M. (2009). Tumor-infiltrating regulatory dendritic cells inhibit CD8+ T cell function via L-arginine metabolism. Cancer Res. *69*, 3086–3094.
20. Marigo, I., Zilio, S., Desantis, G., Mlecnik, B., Agnellini, A.H.R., Ugel, S., Sasso, M.S., Qualls, J.E., Kratochvill, F., Zanovello, P., et al. (2016). T Cell Cancer Therapy Requires CD40-CD40L Activation of Tumor Necrosis Factor and Inducible Nitric-Oxide-Synthase-Producing Dendritic Cells. Cancer Cell *30*, 377–390.

21. Hernandez, C.P., Morrow, K., Lopez-Barcons, L.A., Zabaleta, J., Sierra, R., Velasco, C., Cole, J., and Rodriguez, P.C. (2010). Pegylated arginase I: a potential therapeutic approach in T-ALL. Blood *115*, 5214–5221.

22. Ensor, C.M., Holtsberg, F.W., Bomalaski, J.S., and Clark, M.A. (2002). Pegylated arginine deiminase (ADI-SS PEG20,000 mw) inhibits human melanomas and hepatocellular carcinomas in vitro and in vivo. Cancer Res. *62*, 5443–5450.

23. Ascierto, P.A., Scala, S., Castello, G., Daponte, A., Simeone, E., Ottaiano, A., Beneduce, G., De Rosa, V., Izzo, F., Melucci, M.T., et al. (2005). Pegylated arginine deiminase treatment of patients with metastatic melanoma: results from phase I and II studies. J. Clin. Oncol. *23*, 7660–7668.

24. Cheng, P.N.M., Lam, T.L., Lam, W.M., Tsui, S.M., Cheng, A.W., Lo, W.H., and Leung, Y.C. (2007). Pegylated recombinant human arginase (rhArg-peg5,000mw) inhibits the in vitro and in vivo proliferation of human hepatocellular carcinoma through arginine depletion. Cancer Res. *67*, 309–317.

25. Lam, T.L., Wong, G.K., Chow, H.Y., Chong, H.C., Chow, T.L., Kwok, S.Y., Cheng, P.N., Wheatley, D.N., Lo, W.H., and Leung, Y.C. (2011). Recombinant human arginase inhibits the in vitro and in vivo proliferation of human melanoma by inducing cell cycle arrest and apoptosis. Pigment Cell Melanoma Res. *24*, 366–376.

26. Duperret, E.K., Perales-Puchalt, A., Stoltz, R., G H, H., Mandloi, N., Barlow, J., Chaudhuri, A., Sardesai, N.Y., and Weiner, D.B. (2019). A synthetic DNA, multi-neoantigen vaccine drives predominately MHC class I CD8 $^{+}$ T-cell responses, impacting tumor challenge. Cancer Immunol. Res. *7*, 174–182.

OMTM, Volume *18*

Supplemental Information

Synthetic DNA Delivery of an Engineered Arginase Enzyme Can Modulate Specific Immunity *In Vivo*

Makan Khoshnejad, Alfredo Perales-Puchalt, Yaya Dia, Peng Xiao, Ami Patel, Ziyang Xu, Xizhou Zhu, Kun Yun, Ishana Baboo, Rehman Qureshi, Laurent Humeau, Kar Muthumani, and David B. Weiner

Supplementary Figures:

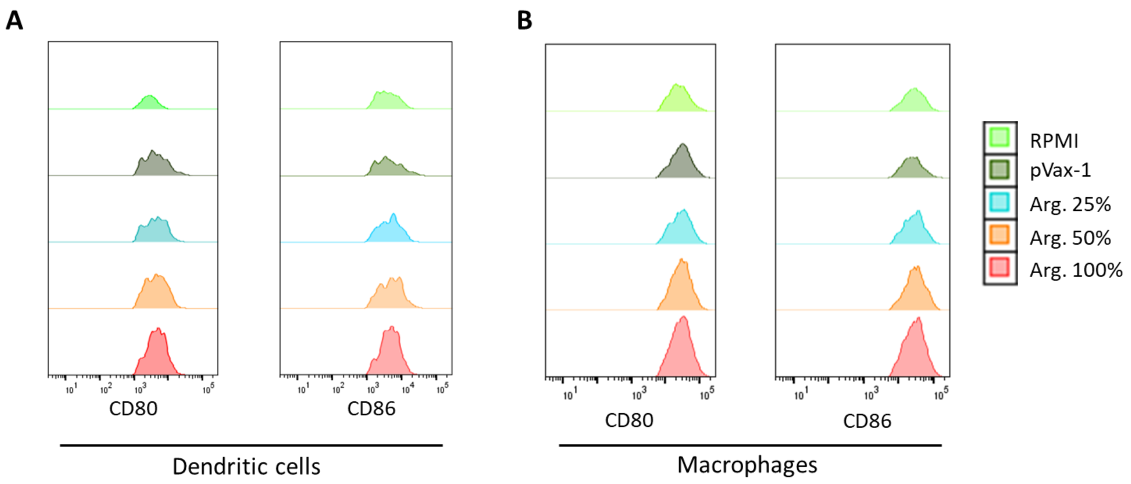

Supplementary Figure 1. Flow cytometry analysis of costimulatory molecules on bone-marrow derived dendritic cells and macrophages. Overlay histograms of co-stimulatory molecule expression in bone-marrow derived (A) dendritic cells and (B) macrophages.
